# Esthetics using monolithic zirconia and hand-layered zirconia fixed partial denture

**DOI:** 10.6026/97320630018651

**Published:** 2022-07-31

**Authors:** S Vaishali, Revathi Duraisamy

**Affiliations:** 1 Saveetha Dental College and Hospitals, Saveetha Institute of Medical and Technical Sciences (SIMATS), Saveetha University, Chennai, India

**Keywords:** Esthetics, fixed partial denture, hand layered, monolithic, zirconia

## Abstract

Edentulism and dental disease have a major effect on the standard of lifetime of patients. Fixed partial dentures have become the treatment of choice for many people for the replacement of edentulous space in the oral cavity. Therefore, it is of interest to correlate and compare the esthetics between monolithic zirconia and hand layered zirconia among fixed partial dentures in Saveetha Dental College. 100 patients who monolithic zirconia and hand layered zirconia had fixed partial dentures were included within the study. Pink and white esthetic scores were evaluated. Data collected were entered in SPSS and analyzed through Chi square test. It was observed that hand layered zirconia have better white esthetic score (p<0.000) and pink esthetic score (p<0.003) when compared to monolithic zirconia fixed partial dentures, which were statistically significant. It was concluded that hand layered zirconia fixed partial dentures have better esthetics than monolithic zirconia fixed partial dentures.

## Background:

Edentulism and dental disease have a profound effect on the standard of lifetime of patients. Tooth loss may be a common finding among individuals [1-2]. Despite the continued progress in oral health services offered worldwide, it has caused a reduced number of partially dentate patients, demanding care actually because it really widened [3-4]. The major patient's complaints are usually associated with compromised oral functions and facial esthetics [5-6]. Dental problems have effects on patient's satisfaction levels with their dentition as they affect esthetics and function [7-8] . Fixed partial dentures (FPDs) have become the treatment of choice for the replacement of edentulous space in the oral cavity thanks to their advantage of being fixed within the mouth and being economical as compared to implants [9],[10]. There are numerous options for prosthodontic replacement of missing teeth like removable partial denture, cast partial denture, fixed partial denture or dental implant [11]. Each prosthesis has its own advantages and disadvantages [12- 13]. Fixed prosthodontic treatment can vary from a restoration of a one destroyed tooth with a crown, replacement of one or multiple missing teeth, or a more sophisticated prosthesis for a multiple number of teeth or for an entire dental arch [14]. Application of ceramic materials is widely utilized in dentistry nowadays. It is primarily used for the fabrication of dental prosthesis and restorations [15- 16]. Due to its superiority in esthetic properties, It is widely used in esthetic dentistry [17-18].Ceramic materials are materials of choice and these are primarily composed of glass ceramics, alumina and zirconia [19-20]. Zirconia was introduced in dentistry during the 1980s. It has good mechanical and chemical properties in comparison to other restorative materials [21-22]. It is widely utilized in dentistry for fabrication of a variety of materials like frameworks, dowels, implants, abutments and orthodontic devices-brackets [23-24]. Its superior esthetic properties in comparison to other prosthetic materials are known. Growing demand for esthetics supported the commercialization of latest metal free and tooth colored restoration which in turn resulted within the growing demand for zirconia prosthesis [25-26]. Dental ceramics has undergone changes in the composition to enhance its strength and potential to face up physiologic occlusal forces. For many years scholars have put effort at a relentless pace so as to seek out a dental material that combines excellent aesthetic characteristics with high mechanical properties. The so-called non-metallic restorations have always represented a challenge for dentistry and only in recent years, with the discovery of zirconia, have achieved this ultimate goal. The main problem that prevented the utilization of metal-free restoration is due to the fact that the ceramic has some micro defects in its overall structure that over long duration they have the ability to widen and in the end lead up to the fracture and therefore the failure and breakage of the prostheses. This was true in case of most of the ceramic materials but not especially for the zirconia which will overcome this problem by transforming from tetragonal phase to the monoclinic phase (t → m) and that allows the dental material to extend greatly in its strength [27]. Therefore, it is of interest to report data on the evaluation of esthetics in monolithic zirconia and hand layered zirconia fixed partial denture.

## Materials and Methods:

### Study Setting:

This study was conducted in Saveetha dental college, predominantly. The advantages of the study include flexibility of the study and less time consumption. The disadvantage of the study include, it is limited to a certain population.100 Fixed dental prosthesis cemented patients were randomly included for the study.

### Sampling:

It is a retrospective study. Data was collected after viewing several patient records and analyzing data of nearly 86000 patients between the months of June 2019 to March 2020.Cross verification of data for errors was done by presence of additional reviewers and by photographic evaluation. Simple sampling was done to minimize sampling bias.

### Data collection:

Data of pink and white esthetic scores of the patients who had received fixed partial dentures with both monolithic and hand layered zirconia in conditions like horizontal and vertical bone resorption in edentulous ridges where implant supported prosthesis was not advised or possible. Data were collected after reviewing case sheets of patients as well as their photographs after FPD fixing. Data was entered in excel in a methodical manner manually and was imported to SPSS. Incomplete or censored data was excluded from study.

### Analytics:

SPSS 2.0 software was used for analysis of data and to obtain results. Independent variables included age, gender and dependent variables included esthetics score (white & pink). Descriptive statistics was used to describe the distribution of age and gender distribution of the population and Chi square test was used to compare between the groups.

## Results and Discussion:

In relation to the age distribution of the fixed dental prosthesis patients, 31-40 years age groups have received the maximum number of fixed partial dentures (38%). Next, is the 21-30 years age group, which constitutes (27%) of the population (Figure 1 - see PDF). In relation to the gender distribution of the study population it was found that out of 100 patients, 56% were males and 43% were females (Figure 2 - see PDF). In relation to the association between type of prosthesis and white esthetic score it was found that hand layered zirconia has no discrepancy in white esthetics, in maximum, which shows that it has better esthetics. The results were statistically significant [Pearson Chi square Value-13.614; p= 0.000 (<0.05)] (Figure 3 and Table 1 - see PDF). In relation to the association between type of prosthesis and pink esthetic score it was found that hand layered zirconia has no discrepancy in maximum in pink esthetic score, which shows that it has better esthetics than monolithic FPD. The results were statistically significant [Pearson Chi square Value-8.746; p=0.003 (<0.05)] (Figure 4 and Table 2 - see PDF). Patients' perceptions of their oral health status and appearance of their prosthesis are important outcomes in prosthodontics. The performance of any fixed dental prosthesis is evaluated by assessing various outcomes of chewing ability, esthetics, duration as well as technical complications. Of this esthetics, plays a vital role, by which success of prosthodontic treatment is determined [28]. In this study we observed that hand layered zirconia FPD has better white esthetic score than monolithic zirconia FPD. We also observed that hand layered zirconia FPD has better pink esthetic score than monolithic zirconia FPD. When the type of prosthetic material was associated with white esthetic score, it was found that it was better for hand layered zirconia FPD. This was similar to the studies by Stawarczyk et al. [29] and Sailer et al. [30]. However, results were contradictory to the studies conducted by Herguth et al. [31]. He stated the monolithic zirconia were better in esthetics when compared to hand layered zirconia. The reasons are opacity, superior strength & durability. Overall literature suggests, white esthetics are best for hand layered zirconia & hence can be implemented in clinical practice. When the type of prosthetic material was associated with pink esthetic score, it was found that hand layered zirconia FPD was better than monolithic zirconia. This was similar to the studies by Kim et al. [32], Pithon et al. [33] and Bomicke et al. [34]. However the results were contradictory to the study performed by Moscovitch et al. [35]. The probable reason could be sample size, geographic location & the lab procedures employed dentist's perception. Overall, hand layered zirconia prosthesis has better esthetics (pink score) when compared to monolithic zirconia. So this can be implemented in clinical practice.

## Limitations:

The limitations of the study include differing sample size, presence of additional examiner to assess aesthetic appearance & other scores to evaluate esthetic other than pink & white esthetic score can be included. The future scope of study is that, since esthetics is a major concern, best prosthetic material to be used, to satisfy the patients needs which results in success of prosthodontic treatment. So hand layered zirconia FPD has proved to be better in esthetics and can be implemented and used widely in future clinical practice.

## Conclusions:

Within the limits of the study, hand layered zirconia fixed partial denture has better esthetics compared to monolithic zirconia fixed partial denture and hence can be widely used in future clinical practice.
